# A New Paradigm for Tissue Diagnostics: Tools and Techniques to Standardize Tissue Collection, Transport, and Fixation

**DOI:** 10.1007/s40139-018-0170-1

**Published:** 2018-04-25

**Authors:** Daniel R. Bauer, Michael Otter, David R. Chafin

**Affiliations:** Roche Tissue Diagnostics, 1910 East Innovation Park Drive, Tucson, AZ 85755 USA

**Keywords:** Tissue fixation, Formalin diffusion, Sample collection, Formaldehyde

## Abstract

**Purpose of Review:**

Studying and developing preanalytical tools and technologies for the purpose of obtaining high-quality samples for histological assays is a growing field. Currently, there does not exist a standard practice for collecting, fixing, and monitoring these precious samples. There has been some advancement in standardizing collection for the highest profile tumor types, such as breast, where HER2 testing drives therapeutic decisions. This review examines the area of tissue collection, transport, and monitoring of formalin diffusion and details a prototype system that could be used to help standardize tissue collection efforts.

**Recent Findings:**

We have surveyed recent primary literature sources and conducted several site visits to understand the most error-prone processes in histology laboratories. This effort identified errors that resulted from sample collection techniques and subsequent transport delays from the operating room (OR) to the histology laboratories. We have therefore devised a prototype sample collection and transport concept. The system consists of a custom data logger and cold transport box and takes advantage of a novel cold + warm (named 2 + 2) fixation method.

**Summary:**

This review highlights the beneficial aspects of standardizing tissue collection, fixation, and monitoring. In addition, a prototype system is introduced that could help standardize these processes and is compatible with use directly in the OR and from remote sites.

## Introduction

Tissue handling for the purposes of downstream evaluation for disease diagnosis consists of two main types of workflow, preanalytics and analytics. Analytics can be loosely defined as the processes that take place after the sample has been collected and is ready to be sectioned for various histological or molecular tests. These workflow steps consist of software that allows a pathologist or oncologist to order specific tests, cutting and microtomy of samples, and staining and pathology review. There has been tremendous effort placed on the analytical side of this workflow where companies have devised automated instruments to accomplish microtomy and staining of the samples. In addition, software solutions exist for tracking patient samples and organizing the various stains that make up a patient’s case. In contrast, much less effort has been placed on the preanalytics aspects of the workflow. Preanalytics for purposes of this article consist of patient admitting to wax embedding. Workflow steps on this side of the equation consist of patient identification and tracking through the hospital setting to sample collection and transport and ultimately embedding the sample into paraffin wax. While there are many different definitions of analytics versus preanalytics, these two workflow processes can have some overlap but can be largely defined as above.

Tissue preanalytics has been a topic of great discussion in recent years due to the fact that these workflow steps are largely non-standardized and many different techniques are considered standard or acceptable within guidelines for use. For instance, each major medical facility might have its own software for accepting patients and for use in the hospital setting. Another example is the use of a wide variety of tissue fixatives, ischemia and fixation times, transport protocols, and paraffin waxes used for embedding. At a minimum, these varied procedures result in tissue blocks that are variable and the data resulting from the use of these blocks might not be reproducible from site to site. An attempt has been made to reduce the preanalytical variation for some biomarkers such as HER2 in breast samples where the American Society of Clinical Oncology/College of American Pathologists (ASCO/CAP) guidelines for HER2 immunohistochemistry (IHC) call for fixation in neutral buffered formalin (NBF) for at least 6 h and no more than 48 h [1, 2]. This still allows for an eightfold variation in fixation time alone.

Poor preanalytics can lead to many errors and inefficiencies in the histology laboratory. These can range from mislabeling patient cassettes [3, 4], improperly fixed (mainly under fixed) tissues [5–8], tissue contamination [9–12], to staining that is compromised [13, 14]. These errors can result in mis-diagnosis, mis-treatment, and costly re-work of patient slides. HER2 the high-profile marker for breast cancer has been at the forefront of many of these investigations. Specifically in Canada, several hundred women tested negative for ER/PR between 1997 and 2005. Laboratory processing errors resulted in re-work of these samples and 40% of 2000 women tested positive, thus making them eligible for the drug tamoxifen. Investigations found fixation and specimen handling as the major problem in histology laboratories [15]. Another high-profile study from MD Anderson Cancer Center found an inconclusive rate for HER2 testing around 10.8%, requiring new samples to be taken. After improving methods to regulate fixation times, the error rate fell to 3.4%, a 64% reduction [16]. Other studies focused specifically on the effects of preanalytics to IHC found 15 preanalytical variables that could have an impact on the stain intensity and quality [17, 18]. It is clear that new preanalytical tools and technologies will be required for standardizing tissue collection and reducing errors in the histology laboratories.

A rapid advancement in the understanding of cancer biology pathways has shifted the pathology field into an era of “personalized medicine” [19, 20, 21•, 22–24]. The field is leaving the era of general diagnosis and going towards treatments based on the specific biology of the tumor. As the number of relevant cancer biomarkers increases and new and specialized drugs are developed, there will be a need for new preanalytical methods capable of standardizing and capturing medically relevant levels of many different types of molecules. Based on literature searches and visits to hospitals and histology laboratories, we have developed a preanalytics program based on the processes that are the least standardized or result in the most errors during tissue collection. We present here a review of that program and some of the tools and technologies that will help to standardize collection, transport, and fixation of tissue samples.

## Tissue Fixation

Modern pathology is built around the principle of preserving tissues such that the in vivo molecular status is maintained at levels representative of the disease state. Tissues are immersed in a solution of fixative which slowly inactivates biological processes, thus preserving the sample. Further processing ultimately allows the tissue to be embedded into wax for thin sectioning and staining for interpretation microscopically. Every year, around 8 billion tissue samples are submitted for processing in the USA alone. With this huge workload, histology laboratories are looking for faster methods of performing fixation, which currently require from several hours to days to complete. Currently, tissue fixation is a series of non-standardized steps from choice of fixative, time in fixative, cold ischemia times, and thickness of tissue all of which could have an effect on downstream assay results [6, 13, 17, 25]. Ideally, this procedure could be standardized and would be quicker with better preservation over a wide range of biologically relevant molecules. One driver of fixation variability in most histology laboratories stems from workflow considerations, where fixation and processing times are selected to fit within a standard workday. Therefore, current preservation methods result in a wide range of tissue quality and new protocols will be required to shift from a workflow-centric process to one in which tissue preservation is optimized for medical content.

The overwhelming majority of histology laboratories use 10% neutral buffered formalin, a standard off the shelf fixative that is inexpensive, and the active component, formaldehyde, has been in use for over a century [26, 27]. Most procedures involving formalin use the fixative at room temperature as a transportation and holding liquid between the operating room and histology laboratory. Use of NBF at room temperature is a time-consuming process taking several hours to days depending on the thickness of the tissue. Other laboratories use a wide variety of fixatives, some based on crosslinking aldehydes and others based on ethanol. The use of different chemicals (aldehydes vs alcohols) has a profound effect on the tissue morphology; one acts by crosslinking proteins and nucleic acids and the other by coagulating cellular components. These very different modes of “fixing” samples will influence the subsequent staining of various targets, for example the hematoxylin and eosin stain (H&E).

The effects of using non-standardized fixation chemicals and protocols can be very costly with unintended effects. Large reference laboratories that are contracted to run standardized staining assays on non-optimized tissue blocks suffer from higher failure rates and can lead to costly re-work. Often, the data generated by one institution is not reproducible at a site running a different fixation protocol thus hampering the ability to compare staining data [18]. Millions of blocks have been produced with widely different fixation protocols and have been archived for future use. Non-standardized practices mean large drug trials are sometimes run with blocks of unknown preanalytical status leading to increased failure rates.

With ever more pressure being placed on histology laboratories to decrease turn-around times, there has been an interest to explore the use of rapid fixation technologies. One such technology being employed is to simply raise the temperature of the fixative to increase the crosslinking rate [28–31]. While this is practically effective, the use of increasing temperatures has led to many reports of unsatisfactory tissue morphology (H&E) and variability in other molecular analysis, including routine IHC stains [31]. Tissues heated in the presence of fixative, which has not had a chance to sufficiently diffuse throughout the tissue, cause two major problems. The first is that formaldehyde crosslinking kinetics increase dramatically and form a crosslinked shell that may prevent or slow further diffusion of the fixative. Second, the tissue is heated in the absence of fixative (especially in the center) which causes cellular enzymes to become more active with resulting cellular degradation. While the outer layers of tissue look properly fixed, the centers of these tissues remain largely unfixed and damaged.

We have previously reported a rapid fixation protocol that is universal for preserving all biomolecules, standardizing workflow and decreasing turn-around time [32]. This was accomplished by employing a two-temperature fixation protocol with standard 10% NBF. In this scenario, tissues are placed in cold formalin (optimally 4 °C). In this first temperature, little crosslinking occurs but diffusion is unimpeded. By cooling the tissues, cellular enzymes are largely inactivated and the cold environment helps preserve biomolecules, especially proteins with post-translational modifications. After a period of cold time, depending on tissue thickness, samples are placed directly into warm formalin (optimally 45 °C) favoring formation of crosslinks. Since formalin is already present throughout the tissue sample, crosslinking occurs evenly throughout the tissue and superior cellular structure is realized. In addition, biomolecules are better preserved due to the almost immediate decrease of cellular enzyme activity. For tissues up to 4 mm in thickness, 2 h of cold followed by 2 h of warm formalin fixation works best. We named this the “2 + 2” fixation protocol or cold + warm (c + w).

Using the 2 + 2 protocol, we initially showed that fixation could be reduced from 24 h (typical room temperature fixation time) to 4 h with this more efficient protocol. Thus, histology laboratories can reduce the urge to rush fixation of samples for workflow considerations. The biggest impact was seen on the increased preservation of post-translational modification of proteins, such as phosphorylation states. Initially, we focused on the highly labile phosphorylated form of protein kinase B, also known as pAKT in a mouse xenograft model system. Subsequently, we extended those results to include 14 phosphorylated targets in human colorectal, breast, and lung carcinoma patient samples collected in a variety of different fixation scenarios [21•, 33••, 34]. We observed an increase in phosphorylated proteins in formalin-fixed paraffin-embedded (FFPE) tissues fixed with the 2 + 2 protocol of 10–50% compared to the same tissues fixed with a standard 24-h room temperature protocol and 20–80% when the tissue had 1 h of cold ischemia before standard room temperature fixation.

**Fig. 1:**
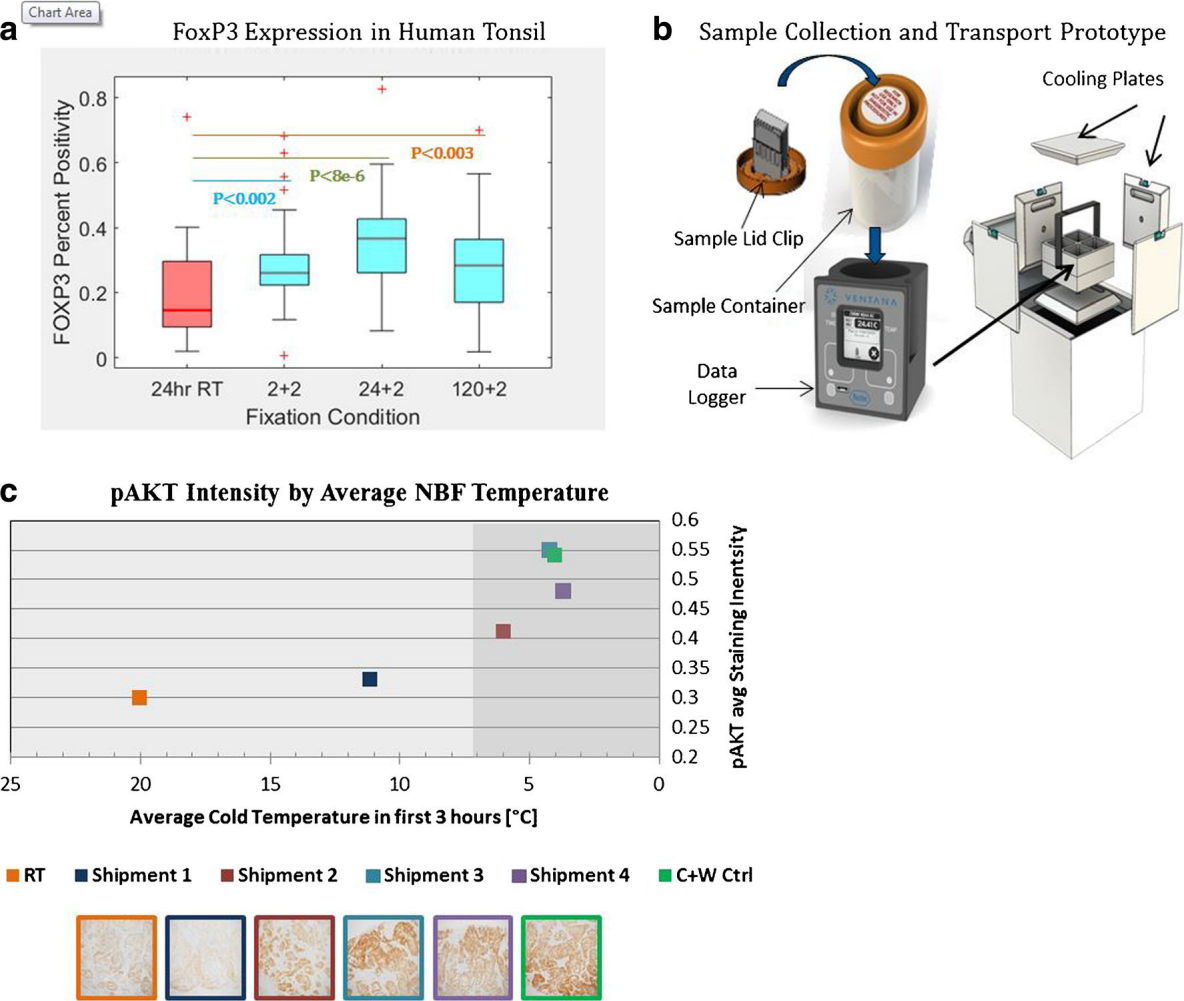
**a** FoxP3 preservation comparing room temperature (RT) and cold + warm fixation protocols using a statistically relevant number of human tonsil samples. Ten whole organs were sliced to 4 mm thickness and randomized. Sixty samples were placed into 4 °C 10% NBF for indicated times (2, 24, or 120 h). After cold soak, samples were then placed into 45 °C 10% NBF for 2 h to complete the crosslinking (samples are referred to as 2 + 2, 24 + 2, and 120 + 2). Alternatively, 18 samples were placed into room temperature 10% NBF for 24 h as comparison (24-h RT). Tissues were stained with an antibody recognizing the FoxP3 protein, digitally scanned and quantitated for expression. On average, cold + warm fixation protocols produced 50–80% more FoxP3-positive cells than the RT protocol (box and whisker graph). The increased detection of FoxP3-positive cells was highly statistically significant. **b** Cartoon depiction of the specimen collection and transport concept to standardize tissue collection and transport. A routine tissue histology cassette is clipped into the lid of a 50-ml standard specimen container. The specimen container contains 10% NBF. The specimen container is then placed into a data logger for tracking of the sample and accurate temperature measurement. Shown here is a prototype data logger from Ventana Medical Systems that measures the temperature of the liquid inside the specimen container and a variety of other parameters. Also shown is a cooling box with inserts to hold multiple specimen containers with data loggers. Cooling panels line the inside of the box and can keep specimens cold for several days. The specimen holder is shown in the middle of the cooling box. **c** Graph relating pAKT staining intensity versus the temperature of the samples in the first 3 h. The graph shows the stain intensity results from 4 separate specimen shipping attempts with room temperature and cold + warm controls. Samples were harvested and sliced to 4 mm thickness. Samples were then loaded into tissue cassettes, placed into 50-ml specimen containers, containers placed into data loggers, and the combination placed into a precooled cooling box for shipment. Once the specimen containers were placed into the data loggers, the data loggers were activated. Once shipped samples were unpackaged, they were placed into 45 °C 10% NBF for 2 h to complete the protocol. Representative pAKT IHC staining results are shown for each of the 4 shipments and the RT and cold + warm controls (IHC pictures, bottom)

More recently, we have focused on the preservation of immunotherapy targets such as the transcription factor forkhead box P3 (FoxP3) and PD-L1. The FoxP3 protein is important for the development and regulation of CD4^+^ immune cells (T regulatory cells). Disruption of the expression of FoxP3 protein, by a number of mechanisms, is correlated with a decrease in the absolute number of T regulatory cells and implicated in autoimmunity [35, 36]. The numbers of FoxP3-positive T regulatory cells are currently being used as a possible prognostic factor in breast cancer, colon cancer, and likely other indications [37, 38•, 39–52]. We therefore undertook a systematic approach to understand the preanalytic sensitivity of this important biomarker to fixation time and protocol, including our novel two-temperature fixation method. Using human tonsil as a model system, we show that 50–80% more positive FoxP3 T regulatory cells are detected when cold + warm fixation is used versus a room temperature protocol with strong statistical significance (Fig. 1a, 2 + 2 vs RT). Interestingly, cold soak times, longer than 2 h, preserve at least as much FoxP3, indicating that the initial cold soak can be used as a holding point for tissues without degradation (Fig. 1a, 24 + 2 and 120 + 2 vs RT).

Another class of biomarkers we examined for preanalytical sensitivity was nucleic acids, both DNA and RNA. The biggest gain in this class of biomarkers was seen with the preservation of long and short RNAs [53]. To study preanalytics on the preservation of RNA, colon, breast, and lung carcinoma clinical patient samples were split into three equal portions and fixed each piece with different protocols. One piece had standard 24 h of room temperature formalin, one piece had a purposeful 1-h cold ischemia then standard fixation, and one piece had the 2 + 2 protocol. Each tissue sample was hybridized, stained for specific RNAs with silver and the slides scanned and the signal was quantitated. In clinical tissues, the amount of detectable long (β-actin) and short (miRNA) RNA species dramatically increased in tissues fixed with the 2 + 2 method (Table 1). A dramatic reduction in RNA levels was observed when the tissues had larger cold ischemia times with standard room temperature fixation (Table 1).

**Table 1:** Fold increase/decrease compared to 24-h room temperature (RT)

Carcinoma type	Cold + warm	24-h RT	1-h Ischemia	Patient ID	
Lung	7.79	1.00	0.07	2	β-Actin
	83.33	1.00	0.33	10	
Breast	1.73	1.00	1.07	4	
	1.72	1.00	0.88	10	
Colorectal	8.40	1.00	0.07	20	
	5.05	1.00	0.01	21	
Lung	1.33	1.00	0.29	6	miRNA21
	1.92	1.00	0.37	10	
Breast	1.27	1.00	0.58	7	miRNA205
	1.28	1.00	1.22	10	
Colorectal	2.24	1.00	0.94	20	miRNA17
	4.08	1.00	0.31	21	

## Diffusion Monitoring

In order to develop faster and more efficient fixation protocols, there needs to be the ability to be absolutely sure fixative has penetrated the sample. There are currently no technologies capable of quantifying the concentration of formaldehyde in tissue specimens. Diffusion of an exogenous chemical into a tissue is a complicated process that is considerably influenced by temperature, tissue heterogeneity, and the molecular size and shape of the penetrating chemical.

Some researchers have soaked tissues in radioactive formaldehyde and used photography film as a measure of diffusion rates while others have used ultrasound (US) to investigate crosslinking by comparing the acoustic properties of samples before and after fixation [54–58]. Ultimately, neither of these techniques would enable real-time monitoring when changes could be implemented to guarantee proper tissue fixation and ideal biomarker preservation.

To address these limitations, we developed a real-time diffusion monitoring system based on measuring the small differences in sound waves, named *T*ime-*O*f-*F*light (TOF) [21•, 59]. As passive diffusion drives formaldehyde into the tissue, the change in the TOF signal manifests as a small acoustic TOF delta that is proportional to the amount of fluid exchange (i.e., ex vivo formaldehyde concentration). This small change in TOF was resolved with digital acoustic interferometry with subnanosecond sensitivity. With this technology, we were able to track and quantify formaldehyde diffusion dynamically until the tissue and bulk fixative became isotonic resulting in maximum formaldehyde concentration. The ability to monitor formaldehyde diffusion can also be used to detect the spatial variability in fixative concentration. We found that diffusion occurs radially from the outside in with the center of the tissue requiring significantly longer to become adequately fixed. The diffusivity coefficients calculated with our system were well-correlated with the apparent diffusion coefficients (ADC) of water measured using MRI that indicated orthogonal validation of our method [21•, 59].

Ultimately, the TOF method was validated on samples from 34 different clinically relevant tissues. The system produced a range of diffusion rates dependent on tissue type and readily identified tissues which are known to require long fixation times (brain and adipose) (Table 2). There was a large variance in decay constants within tissue types, however, indicating that associating a single optimal fixation time to any given tissue type would not be prudent. Using TOF, we observed heterogeneous formalin diffusion even within a single piece of tissue and further exacerbates the need to treat each specimen individually when considering formalin fixation. Our developed diffusion monitoring technology provides a quantifiable parameter of tissue fixation that will enable the effects of key preanalytical variables on downstream assays to be studied. This technology could enable truly personalized processing of individual biospecimen by ensuring each tissue gets the optimal amount of formaldehyde.

**Table 2:** Diffusivity constants for normal human tissues

Tissue type	Decay constant	Stnd dev.	Diffusion rate
Artery	0.54	Na	Rapid
Gallbladder	0.81	0.36	
Muscle	1.01	0.21	
Lung	1.06	0.32	
Ovary	1.08	0.53	
Kidney	1.13	1.21	
Pancreas	1.13	0.38	
Ribcage	1.14	0.15	
Stomach	1.16	0.58	
Rectum	1.19	0.44	
Prostate	1.27	0.07	
Jejunum	1.33	2.47	
Cervix	1.36	0.12	
Colon	1.37	0.00	
Breast	1.38	0.05	
Testis	1.40	0.23	
Thyroid	1.40	0.86	
Ileum	1.46	0.21	
Uterus	1.56	0.31	Medium
Bladder	1.68	0.23	
Appendix	1.69	0.13	
Adrenal gland	1.75	0.16	
Esophagus	1.85	0.19	
Tongue	1.90	0.04	
Lymph node	1.96	0.50	
Duodenum	2.02	1.65	Slow
Cardiac	2.09	0.56	
Brain	2.56	0.29	
Skin	2.60	0.25	
Tonsil	2.75	0.23	
Liver	3.23	Na	
Spleen	3.36	0.54	
Fat	3.69	0.04	

## Cold Collection and Transport

The last important area we have addressed is the collection and transport of tissue samples from the OR to the histology laboratory. There are two main concerns which affect downstream assay results: minimizing the amount of time samples sit at room temperature before proceeding to the histology laboratory (cold ischemia times) and controlling physical parameters such as transport time and temperature. Offering a transport solution allows samples collected at satellite sites to also have careful collection protocols during transport to larger processing facilities. To this end, Ventana Medical Systems Inc. (VMSI) has developed a prototype for shipping fresh, unfixed tissue samples that is compatible with the 2 + 2 (cold + warm) fixation protocol (Fig. 1b). The prototype consists of a cooling box designed to remain “cold” for 3–5 days, a custom data logger that is designed to hold a standard specimen container and custom foam pieces to minimize impact to the components. The prototype once loaded with tissue samples can be placed inside a standard cardboard box and shipped through a commercial source (such as FedEx).

In order to validate that tissue samples could be collected and shipped in this manner, we shipped four different sets of Calu-3 mouse xenograft tumors from Tucson, AZ, to Seattle, WA, and back via FedEx. Calu-3 xenografts were used as this tumor produces high levels of phosphorylated-AKT protein and has been shown by us and others to be highly labile and difficult to preserve with standard room temperature fixation techniques [34, 60]. Since we were learning how to collect, package, and ship samples, the first two shipments resulted in samples that had compromised pAKT staining due to the temperature of the formalin drifting too high during the first hours after collection (Fig. 1c). In subsequent shipments, we were able to maintain temperatures below 7 °C by using refrigerated formalin and precooling all components (Fig. 1c, shipments 3 and 4). When temperatures were kept below 7 °C, we were able to maintain staining levels of pAKT identical to staining controls which consisted of half of the tumor being preserved immediately by the 2 + 2 method (Fig. 1c, c+w ctrl). In conclusion, we have demonstrated an easy to use collection and transport concept that can be implemented in a hospital setting that could help to standardize tissue collection and transport and offer a tracking solution for patient safety that does not exist currently.

## Conclusions and Discussion

By utilizing primary literature and customer data, we have identified the preanalytical areas that are most relevant to preventing errors due to specimen collection, handling, and tracking. We have developed a suite of new technologies that will help to standardize the collection, fixation, and transport of tissue samples from hospital and clinical settings.

One potential benefit from the use of cold collection is to standardize tissue collection and decrease the time of fixation. Most histology laboratories collect and process samples based on workflow considerations and the use of batch automated equipment. As a consequence, samples need to be held in liquids or placed onto processors with very little fixation time, leading to downstream assay variability. We have developed a rapid fixation method and other tools to lessen the likelihood that technicians will need to rush these processes. In addition, the 2 + 2 fixation method has been shown to improve preservation of a wide variety of important biomarkers from post-translational modifications, routine IHC markers, and nucleic acids making this an attractive solution in the future.

Our developed diffusion monitoring technology provides a quantifiable parameter of tissue fixation that will enable the effects of key preanalytical variables on downstream assays to be studied. This technology could enable truly personalized processing of individual biospecimens by ensuring each tissue gets the optimal amount of formaldehyde. A next-generation tissue processor based on this technology would expedite tissue-processing times and standardized tissue fixation in a fully traceable preanalytics workflow.

Traceability of a patient’s sample from origin through the histology laboratory and ultimately to case sign out is becoming a discussion point for standardized patient care. We have shown results for a prototype data logger that would encompass the sample and record various parameters. The most salient feature is the ability to record the temperature of the fixative while the sample is either being shipped, transported, or stored. We have found that the most important time for the 2 + 2 method is keeping the formalin below 7 °C during the first 3 h. Knowing this information can alert the medical team that samples either qualify or do not for the next advanced biomarker tests that rely on careful collection techniques. In addition, this data logger is able to record if a sample is present, if the main collection box has been opened and compromised, and if the samples have received any physical shocks. While some of these features may only be useful for prototype development, having the ability to track and trace a sample through the process is very attractive.

In addition to shipping samples using the sample collection and transport concept, we have recently explored the ability of using this technology directly in an OR setting. The individual components have been engineered to be decontaminated and re-used multiple times. In collaboration with the University of Washington, we collected 50 samples from metastatic liver tumors that were removed, examined, and placed directly into cold formalin in the OR. Patient samples were placed in a cassette, clipped into the lid of a standard specimen container, placed into a waiting data logger, and then transported from the OR in a cold storage box.

One area of great interest to the pathology field is the use of digital equipment to scan and interrogate stained slides. An increasing number of digital algorithms are getting FDA approval, one example being staining of breast tumors for the overexpression of the HER2 protein. The use of these algorithms for possible diagnosis and treatment will require standardized tissue collection and staining. The highest hurdle is the fact that under-fixed tissue samples exhibit weak staining signals. The use of a collection and transport system would be the first step in ensuring tissue is of the highest quality.

Tissue collection is a non-standardized process that is largely built around the workflow of the histology laboratory. Very little of what happens to the tissue sample, after excision, is based on biomarker preservation, quantitative parameters, or scientific principles. We have designed our program and hence the tools and technologies under the guidance of making a science of tissue collection. Precision medicine for patients will require a deeper understanding of the biology that drives tumor development and growth. With standardized collection and transport processes, new classes of biomarkers can be preserved with greater efficacy than has been realized in the last century.
